# A Fatal Case of Cor Pulmonale with Undetected Chronic Hypoventilation in an Infant with a Known Congenital Myopathy

**DOI:** 10.1155/2012/836420

**Published:** 2012-06-03

**Authors:** John M. Holst, Mary J. Willis

**Affiliations:** ^1^Department of Aviation Medicine, Naval Air Station North Island, Parkville, MD 21234, USA; ^2^Department of Genetics, Naval Medical Center San Diego, San Diego, CA 92134, USA

## Abstract

The authors of this paper wish to present a case of fatal cor pulmonale with right ventricular hypertrophy complicated by a congenital myopathy. It is our intention to demonstrate the importance of vigilant clinical assessment of children with a congenital myopathy, regardless of the exact etiology of their disease, or family history of disease severity. This case highlights the risk for fatal complications if hypoventilation and respiratory insufficiency go unrecognized in myopathic children. Consequently, we recommend respiratory and cardiac monitoring surveillance as well as appropriate referral to specialists in the management of such children.

## 1. Introduction

Congenital myopathies consist of a heterogeneous group of neuromuscular disorders that normally present at birth or early childhood. The inherited muscle disorders are defined by distinctive histochemical and ultrastructural changes in the muscle. They present with hypotonia, weakness, and usually delayed motor milestones [1]. The phenotypic presentation and natural history of these disorders can be quite variable, even among affected individuals from the same family. 

There is no cure for congenital myopathies, and optimal management requires a multidisciplinary approach, particularly as it pertains to their respiratory status. We present the case of an infant who was the third generation in his family affected by a congenital myopathy in which cor pulmonale developed undetected. The fatal outcome of this case illustrates the critical importance of vigilant monitoring of the respiratory and cardiac status during every patient encounter in a child diagnosed with a congenital myopathy.

## 2. Case Report

This patient was a Caucasian male infant, born vaginally at term without any prenatal complications, who presented with severe hypotonia immediately after delivery. His birth weight was 6 pounds 7 ounces. The infant did not require intubation, nor did he demonstrate signs of severe respiratory distress at birth. He displayed a high-arched palate, tented upper lip, pectus excavatum, and bilateral cryptorchidism. Ophthalmoplegia and bulbar weakness were not noted in this patient. His severe hypotonia was more prominent in his upper extremities compared to his lower extremities, while his reflexes were absent throughout. 

Family history was significant; his mother and maternal grandmother were diagnosed with a congenital myopathy. His mother was born with hypotonia and diagnosed with type II congenital fiber type disproportion (CFTD) at 18 weeks of age following a muscle biopsy. Subsequently, the maternal grandmother, who also displayed mild muscular weakness, had a confirmatory muscle biopsy consistent with her daughter's results. The family admitted to global hypotonia, with involvement greater in the upper extremities than in the lower, but not to the severity of this patient. 

The infant's evaluation while in the neonatal intensive care unit (NICU) included normal chromosomes, fluorescence in situ hybridization (FISH) for Prader-Willi, and very long chain fatty acids. He had normal creatine kinase with an elevated MB fraction. Ultrasound studies of the head, kidneys, and heart were all negative. His chest X-ray did demonstrate a “bell-shaped” chest with an elevated right-sided hemidiaphragm, without evidence of any scoliosis or other orthopedic abnormalities. Genetic testing for myotonic dystrophy was performed on the patient's mother and was negative. He was given a presumed diagnosis of CFTD consistent with his family history. The patient was without apneic events for the three weeks he was in the NICU, and the cardio respiratory gram (CRG) was negative. Due to the concern for positional obstruction, he was discharged to home with a home inductance plethysmography monitor that sensed his respiratory movements and his heart rate but not his oxygen saturation. 

As an outpatient, the parents admit that the home respiratory monitor alarm sounded a few times but was limited to car seats or other carrying devices consistent with the concern for positional obstruction; repositioning of the infant corrected the situation without further concern. The monitor was used regularly except during brief times the child was awake, feeding, or bathing with the parents. The greatest concern for outpatient medical management of this patient was his poor weight gain. After 10 weeks, he had only gained 600 grams after incrementally increasing his fortified breast milk each week to the maximum 27 calories per ounce. He was well below the 5th percentile for weight, and his height percentile was 75. With the increased supplementation, he was gaining on average 15 grams per day which was the minimum goal set by the pediatric gastroenterologist. The parents admitted that feedings were very strenuous for the infant, and the plan from the pediatric gastroenterologist was to place a gastrostomy tube once he reached 5 kilograms; at that time his formal muscle biopsy would be performed as well. 

At 14 weeks of age, the patient developed respiratory failure and died unexpectedly. At autopsy, the cause of death was identified as cor pulmonale with marked right ventricular hypertrophy (RVH) complicated by congenital myopathy. He weighed 9 pounds 7 ounces at the time of his death. His heart weighed 42 grams (expected weight of 30 grams), the right ventricle measured 1.0 cm in thickness and was bulging (expected 0.25 cm), and the left ventricle, valves, and coronary arteries failed to demonstrate any congenital abnormalities. The respiratory system demonstrated atelectasis throughout the lungs, with an obvious bell-shaped chest with pectus excavatum, and the lungs weighed 57 grams total (mean 89 grams) and failed to demonstrate any gross consolidations, tumors, abscesses, or infarcts. The liver displayed normal size with centrilobular congestion and necrosis of the liver. 

The skeletal muscle demonstrated marked variation in fiber size with some very small atrophic muscles. There were a few central nuclei, but this was not a prominent finding. No central cores and no inflammation, fibrosis, or other structural abnormalities were seen besides the obvious disproportion between muscle fibers. The type II fibers measured much smaller than the type I muscle fibers. The histopathologist gave a diagnosis of type II CFTD, consistent with a previous family diagnosis. The rest of the autopsy failed to demonstrate any other findings or abnormalities. 

## 3. Discussion

The case of presumed autosomal dominant congenital myopathy presented here highlights the point that congenital myopathies are not always benign, and in some cases they possibly are progressive [2]. As an infant, the mother was more affected than the grandmother, which prompted the initial investigation for a congenital myopathy in the grandmother at the age of 30. Consistent with his mother and grandmother, the infant's muscular weakness was more significant in his upper extremities than in his lower extremities. While it was obvious the patient here demonstrated increased muscle involvement compared to the mother and grandmother, he failed to demonstrate respiratory distress while in the NICU or after his discharge until his unexpected death. His cardiorespiratory status appeared benign and stable, although he did display difficulty in gaining weight. He was well below the 5th percentile for his weight but did demonstrate consistent gains on his growth curve after increased supplementation, albeit well below normal. 

The authors of this case report hypothesize that the infant, while not displaying respiratory insufficiency in the NICU for the first three weeks of life, developed chronic hypoventilation that was not detected, even with the home inductance plethysmography monitor. The chronic hypoventilation was most likely due to muscular weakness evidenced by pulmonary atelectasis and the bell-shaped chest on autopsy. Ward et al. state that rapid skeletal growth spurts may outpace the respiratory muscle capacity to maintain appropriate ventilation, thus causing indolent respiratory decompensation leading to undetected respiratory failure [3]. If respiratory decompensation leading to hypoxemia and hypercapnia is not corrected, it may blunt the peripheral and central chemoreceptor responses, further aggravating the chronic alveolar hypoventilation [4]. His echocardiogram was devoid of any evidence of cardiac abnormalities during his first week of life, but within 3 months, the cardiac hypertrophy and chronic hypoventilation developed undetected during routine pediatric and specialist visits. 

According to Clarke and North [5], there may be some debate about the legitimacy of the diagnosis of a type II CFTD, but after careful review of the case, the child clearly falls under the broader category of a congenital myopathy [5]. As noted by Petterson et al., monitoring or surveillance of children with neuromuscular disorders (NMD) is extremely important because respiratory failure is often progressive and can go undetected, with fatal consequences [6]. The literature concerning CFTD and other congenital myopathies over the years has clearly identified cases that are not benign; those with severe muscle involvement usually demonstrate severe respiratory impairment. However, the severity of the respiratory and limb involvement can be discordant, so clinical exam alone may not be the optimum guide to suggest further investigation. In some cases, respiratory failure can occur at any age and without evidence of superficial respiratory distress, as demonstrated in this case [4, 7]. Clinical evidence is lacking in surveillance techniques and protocols tailored to specific neuromuscular diseases, particularly with congenital myopathies, due to the sparse data for the natural disease progression [6]. It is imperative for the general pediatrician to refer these patients early to the appropriate pediatric specialists not only to improve their quality of life but also to influence their survival [2].

Current recommendations are that all pediatric patients with a diagnosis of a congenital myopathy should have a baseline pulmonology evaluation [2]. Surveillance by a pediatric pulmonologist should be biannual at least, to concentrate on regular age-appropriate pulmonary function testing, particularly for decreased nocturnal ventilation with consideration for early polysomnography and lung function tests to include supine vital capacity and arterial blood gases [6]. Further pediatric subspecialties should be involved to include neurology, pulmonology, cardiology, and any other clinically warranted specialists. Routine echocardiograms and electrocardiograms (EKGs) should be performed at baseline and then as clinically indicated for most congenital myopathies. In his review article from 2003, Muntoni recommends that CFTD myopathies should have EKGs and echocardiograms at baseline and every 5 years after that, or sooner if clinically indicated [8].

The authors of this paper would like to argue that, based on this case, a more aggressive surveillance of the respiratory system, as well as the cardiac system, may be warranted during the first year in patients with a congenital myopathy. Due to the benign family history and reassuring initial respiratory and cardiac evaluations in this case, more aggressive surveillance was not implemented, nor were cardiac and respiratory pediatric subspecialties involved in the outpatient management of this patient. This patient developed undetected severe RVH in a remarkably short period of time, less than 3 months. Early identification of respiratory decompensation is crucial so that noninvasive ventilation (NIV) can be implemented as early as possible to prevent further respiratory and cardiac degradation and possibly reverse it with appropriate respiratory therapy [6]. If the patient is less than 5 years old or vital capacity cannot be evaluated, nocturnal oximetry and transcutaneous carbon dioxide is an acceptable evaluating tool that should be performed at least annually [4, 6]. Home monitoring systems that evaluate oximetry could be used as appropriate screening tools for chronic hypoventilation in young children with congenital myopathies, in addition to routine polysomnography testing in accordance with the pediatric pulmonologist's recommendations. In 2005, Hammer and Eber identified failure to thrive as a common indication for undetected hypoventilation with children diagnosed with congenital myopathies [9]. They also present an excellent detailed summary of signs and symptoms of sleep-disordered breathing (SDB) and respiratory impairment symptoms, which should be specifically sought and documented during every pediatric visit [6].

To our knowledge, we present the youngest fatal case of cor pulmonale with right ventricular hypertrophy complicated by a congenital myopathy. After reviewing the literature on congenital myopathies, it is clear this is not the first fatal case of an infant to have hypoventilation with cardiac and respiratory involvement, nor severe complications, but this case highlights the possibility of rapid transition from benign respiratory and cardiac status to severe cardiac and respiratory impairment [10–20].

It is unclear if this case was exceedingly unusual or if this case or cases like it are just underreported in the literature or un/underrecognized. In 1997, Danon et al. described the unexpected death of a twin with a known congenital myopathy at 1 year of age attributed to respiratory complications, but no autopsy was performed to clearly identify the cause of death [2]. Two other cases have been reported in which patients developed cor pulmonale complicated by a congenital myopathy as in this case, but the patients were 9 and 14 years old at the time of diagnosis [11, 20]. Regardless of whether this case is exceptionally rare or represents underreported fatalities that were attributed to respiratory complications or other causes, it is evident that chronic hypoventilation in infants with congenital myopathies can create severe consequences. 

Further studies and investigation of congenital myopathies are required to clearly identify each disease process and be able to risk stratify, allowing for specific disease monitoring and surveillance protocols for each subtype of congenital myopathies. Until then, the authors of this case report illustrate the ramifications of treating congenital myopathies as a benign disease. It is the authors' hope that this case report will demonstrate to the pediatric generalist managing such cases the need to consider the above recommendations for more vigilant pulmonary and cardiac surveillance. In addition to the above recommendations, subspecialty consults of pediatric cardiology and pulmonology should be consulted early to assist in the care of these phenotypically diverse patients. It is imperative that the doctors taking care of these patients understand that a benign appearing clinical exam in a child with a congenital myopathy does not eliminate the possibility of respiratory compromise. Vigilant surveillance is necessary to avoid fatal consequences. 

## Abbreviations

CRG: Cardio respiratory gram
CFTD: Congenital fiber type disproportion
EKG: Electrocardiogram
FISH: Fluorescence in situ hybridization
NMD: Neuromuscular disorders
NICU: Neonatal intensive care
NIV: Noninvasive ventilation
RVH: Rightventricular hypertrophy
SDB: Sleep disorder breathing.
